# An alternative approach to treatment of inferior vena cava filter perforation

**DOI:** 10.1590/1677-5449.180131

**Published:** 2020-05-08

**Authors:** Mariana Krutman, Guilherme Yazbek, Kenji Nishinari, Bruno Soriano Pignataro, Guilherme Andre Zottele Bomfim, Rafael Noronha Cavalcante, Guilherme Centofanti, Igor Yoshio Imagawa Fonseca

**Affiliations:** 1 A.C. Camargo Cancer Center, São Paulo, SP, Brasil.

**Keywords:** deep venous thrombosis, pulmonary embolism, inferior vena cava filter, trombose venosa profunda, embolia pulmonar, filtro da veia cava inferior

## Abstract

We report a case of inferior vena cava filter perforation immediately after filter implantation, recognized intraoperatively in a patient undergoing laparotomy for resection of locally advanced ovarian cancer. We describe an alternative approach with strut resection, less invasive than filter removal, enabling the device to be maintained and bleeding to be controlled.

## Resumo

Relatamos um caso de perfuração de veia cava inferior imediatamente após o implante de um filtro. A complicação foi reconhecida no intraoperatório de uma laparotomia para ressecção de um câncer de ovário localmente avançado. Descrevemos uma abordagem alternativa, menos invasiva do que a remoção do filtro, consistindo na ressecção das hastes do dispositivo. Essa abordagem permitiu a manutenção do filtro e o controle efetivo do sangramento.

## INTRODUCTION

Pulmonary embolism remains a significant cause of mortality and morbidity in cancer patients despite increased use of deep venous thrombosis (DVT) prophylaxis.^1^

Inferior vena cava (IVC) interruption is indicated in patients with venous thromboembolism (VTE) and contraindication for anticoagulation or in cases of recurrent pulmonary embolism despite adequate anticoagulation. This procedure may present complications such as: filter migration, fracture, vena cava thrombosis, and perforation of neighboring structures. Filter perforation can be especially serious when the surrounding tissues affected are the aorta, portal vein, renal vein, liver, kidney, or bowels^2^^,^^3^.

We present a case of IVC perforation that was identified during a laparotomy and treated without filter removal. The technique described avoided a procedure involving greater morbidity requiring vein clamping, cavotomy and filter removal.

## CASE REPORT

A 54-year-old female patient with a personal history of 2 pregnancies and a family history of breast cancer (aunt and two cousins) presented at the emergency department with metrorrhagia and abdominal pain. Complementary imaging investigation with magnetic nuclear resonance revealed a 12x7x12 cm ovarian cystic lesion with internal septations and solid portions, suggestive of ovarian cancer. Moderate ascites and nodules on the peritoneal surface (subphrenic and left parietal-colic gutter) were also observed.

During the preoperative period, the patient presented left lower limb pain and was diagnosed with proximal DVT of the popliteal and posterior tibial veins. Full dose anticoagulation was immediately initiated with enoxaparin.

Despite the acute DVT and in agreement with the oncology surgical team, the patient decided not to delay the proposed aggressive surgical intervention. Given the need for surgical intervention in the setting of an acute proximal DVT, we indicated implantation of a removable vena cava filter.

Implantation of a Bard G2X inferior vena cava filter was performed as the initial procedure, before the laparotomy. The vascular intervention was uneventful, with a right femoral vein access for filter implantation and deployment at the level of the third lumbar vertebra (L3). The subsequent oncologic procedure was performed by means of a xipho-pubic median laparotomy, followed by tumor cytoreduction with total abdominal hysterectomy, bilateral salpingo-oophorectomy, pelvic lymphadenectomy, omentectomy, retroperitoneal biopsy, and inter-cavoaortic lymphadenectomy.

**Figure 1:**
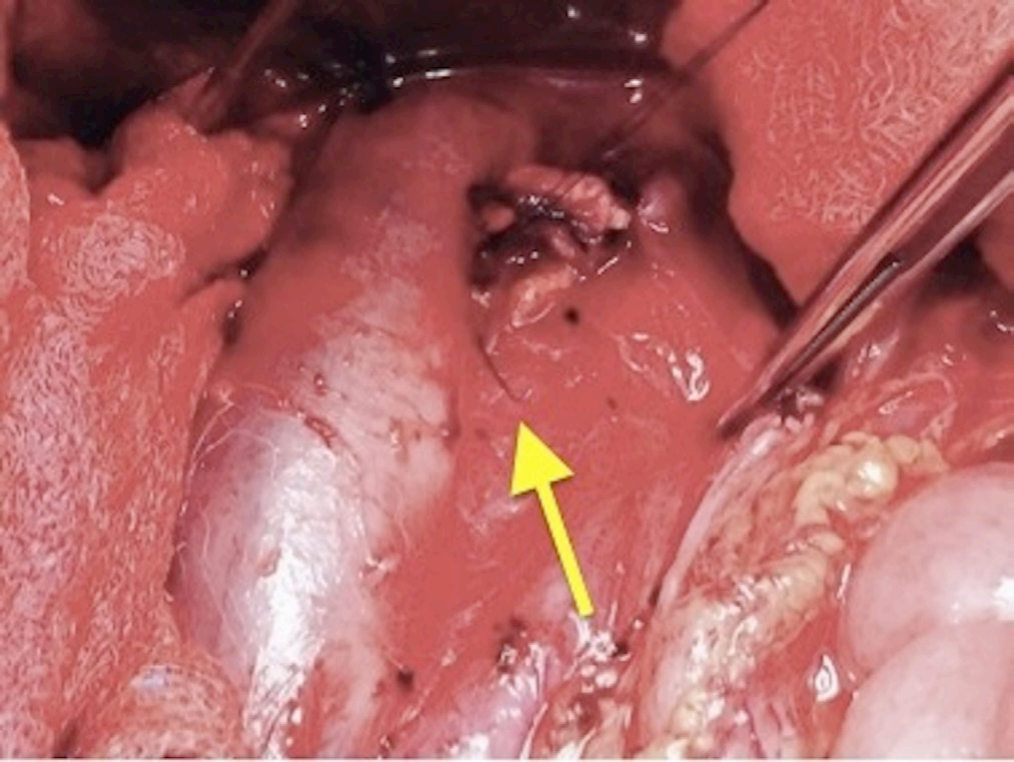
Perforation of the anterior medial wall of the IVC by the filter strut (arrow).

During the tumor dissection and lymphadenectomy, bleeding from the IVC was observed. While attempting to control hemorrhage by suture of the vena cava, the metallic structure of the filter strut was observed perforating the cava wall. After further dissection, three perforations of the IVC were detected at the level of the filter placement. One of the perforations was anterior to the vena cava wall, the other two were in a lateral and posterior position (Figure 1).

**Figure 2:**
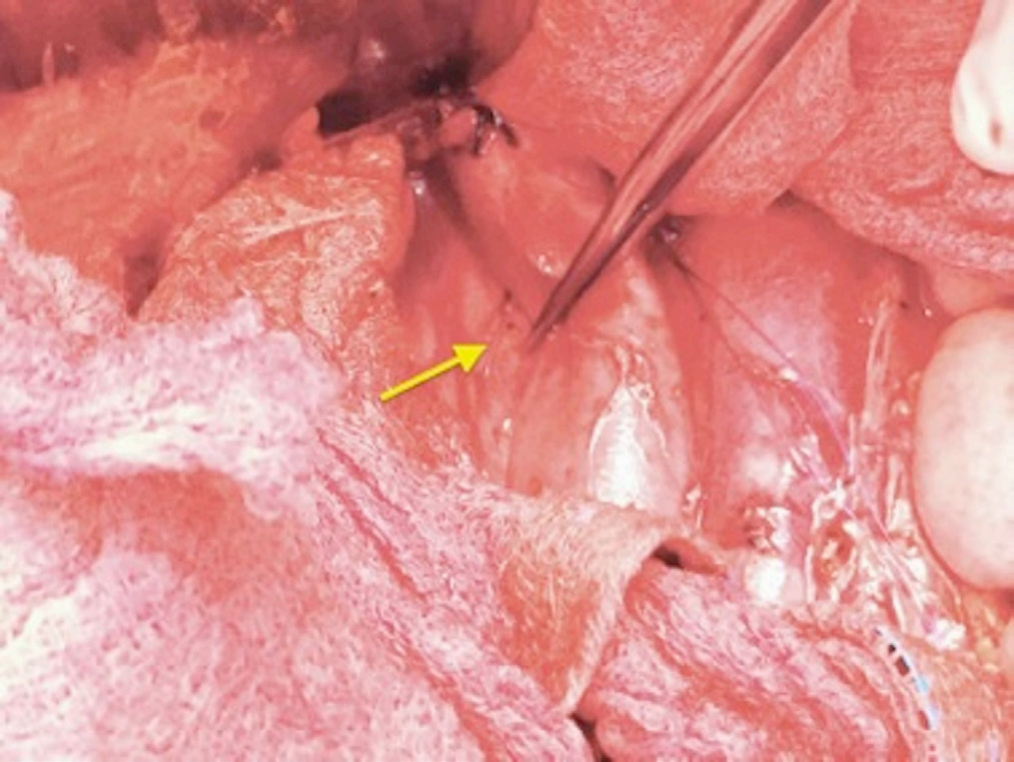
Filter strut perforating the posterior wall of the IVC (arrow), anchored to the surrounding paravertebral tissues.

The posterior metallic strut was anchored and fixed to paravertebral tissues posterior to the IVC and presented no signs of active bleeding or hematoma (Figure 2).

**Figure 3:**
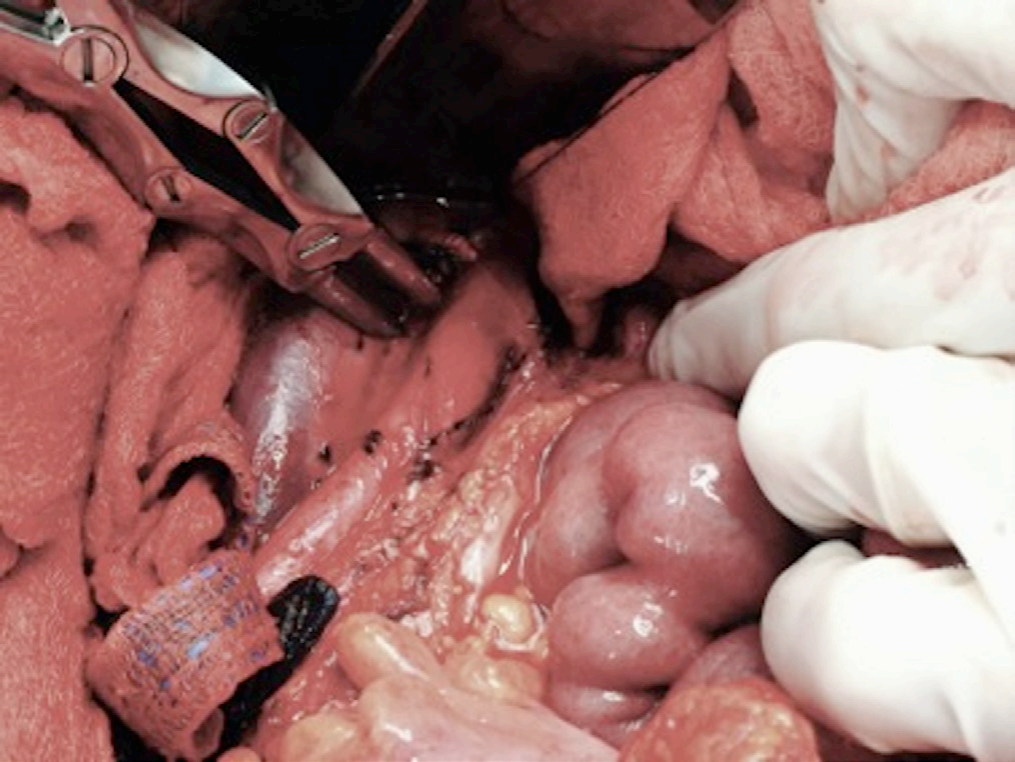
Sectioning the filter strut perforating the anterior wall of the IVC.

**Figure 4:**
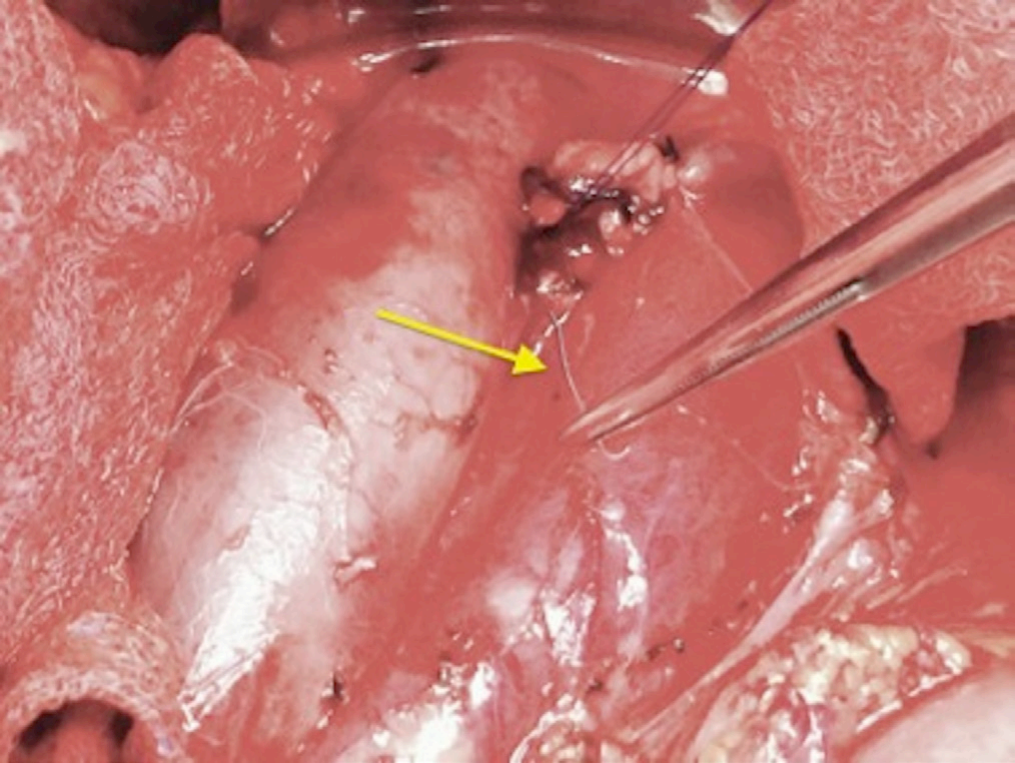
Filter strut (arrow) being removed from the operating field.

After suture of the anterior vena cava wall, enabling control of the main bleeding point originating from the perforation, we decided not to perform a cavotomy for filter removal due to the high risk of further hemodynamic instability in an already critical situation. We chose instead to resect the filter struts that penetrated anteriorly and medially the IVC wall, using Liston shears (Figure 3 and 4). The filter remained in the same position after sectioning of the struts and the abdominal wall was closed.

The patient was discharged on the 10th day after the procedure. During follow-up, anticoagulation was resumed and the patient showed no signs of lower limb edema 2 months after surgery. A control abdominal computed tomography performed after surgery showed vein patency and no filter migration.

## DISCUSSION

Since the first IVC filter devices were described by Greenfield in the 1970s, increased implantation over subsequent decades has culminated in occurrence of complications. Complications related to IVC filters can be divided into three main categories: risks associated with filter insertion; risk of device failure; and risks of long-term complications arising from the filter device itself,^4^ including perforation of the IVC and surrounding tissues such as the aorta, portal, and renal veins, vertebral body, kidney and liver parenchyma, duodenum, large intestine, diaphragm, urinary tract, and retroperitoneum. Patients may present with silent retroperitoneal hematoma, sepsis, and gastrointestinal bleeding or can develop other symptoms related to the injured organ or structure.^2^

The incidence of perforation of the IVC wall has been reported as about 0.2% of patients who underwent Greenfield filter placement and duodenal perforation has been reported repeatedly.^5^ In these cases, clinical findings usually include gastrointestinal symptoms and diagnosis is confirmed with complementary examinations, such as upper gastrointestinal endoscopy, abdominal tomography or MRI.

Radiographically, as many as 25% of IVC filters perforate the IVC wall although the precise mechanism of penetration is poorly understood. In our case, intra-operative surgical manipulation may have contributed to this complication, especially during the inter-cavoaortic lymphadenectomy.

Complementary imaging exams are usually necessary to detect strut perforation, especially in asymptomatic patients. In the case reported, complementary examinations were unnecessary because the complication was detected intraoperatively due to bleeding from the IVC.

In a review by Malgor and Labropoulos,^6^ the most frequent type of filter causing duodenal perforation was the Greenfield filter (Boston Scientific Corp, Natick, Mass), followed by the Bird’s nest filter (Cook, Bloomington, Ind), and the Mobin-Uddin (no longer sold). However, other filters such as the Recovery filter (Bard Peripheral Vascular, Tempe, Ariz), the Celect filter (Cook), and the Gunther-Tulip filter (Cook) have also been linked with this complication. In our case, the G2X (Bard) filter was used. Duodenal and aortic perforations have been described after implantation of this device as well as after implantation of the others mentioned above.^7^

Filter fracture is a complication described in 14% of Cordis OptEase and TrapEase filters in up to 4 years post-IVCF implantation, as reported by Wang et al. The same authors described complete or partial IVC occlusion in 13% of cases (7.3% total and 5.2% partial) and higher rates of IVC perforation for retrievable conical type devices (70%) compared with permanent devices (15%), especially involving retroperitoneal structures when conical retrievable devices were used.^8^

In cancer patients, insertion of a vena cava filter is only indicated for patients with contraindications to anticoagulant therapy, as described in this case, surgery or invasive procedure within 1 month of surgery. It remains unclear whether permanent or retrievable filters are preferable in the cancer setting. It is reasonable to select a retrievable filter when the contraindication to anticoagulation is expected to be transient, even though serious concerns have been raised with relation to the safety of retrievable filters.^9^ At our service, we have decided to preferentially fit retrievable filters in situations such as that described in this case and remove them postoperatively.

Open surgery to manage symptomatic patients with filter-related IVC perforations can be challenging. The need to expose the IVC involved in an inflammatory reaction, followed by clamping and sectioning of the vein are technical aspects that add to the increased morbidity of this approach.

Filter removal using an endovascular approach has been reported, with the advantage of reduced morbidity when compared to the open procedure. However, in a systematic review of duodenal filter perforation, only one of the 21 cases analyzed was treated using the endovascular approach.^10^ The endovascular technique requires a new access route and involves a risk of laceration of the vena cava wall at the time of filter capture.

Filter migration is a complication that should be considered after removal of the fixation struts. However, in this particular case, the posterior strut that was observed to be anchored to paravertebral tissues minimized the risk of this problem.

Although removal is recommended for filters with complications, reports have been published describing conservative management with rigorous clinical and radiological surveillance.^6^ In our case, a more conservative initial approach was chosen with the plan to remove the filter at a later date.

## CONCLUSION

Although IVC filters remain a safe and excellent method to prevent life-threatening PE in situations where anticoagulation is contraindicated, complications such as perforation can be critical. Correct indication of filter implantation, patient follow-up with a view to future retrieval, and improvements in device design are factors that can minimize complications. We describe a more conservative approach with strut resection that enabled the device to be maintained and bleeding to be controlled. The filter described in this case has been withdrawn from medical use due to a high incidence of complications. Since then, our service has stopped using this device, choosing other temporary filter models.
